# Evaluation of patients with severe pulmonary hypertension and a range of comorbidities prescribed inhaled treprostinil

**DOI:** 10.1016/j.jhlto.2024.100131

**Published:** 2024-07-23

**Authors:** Aparna C. Swaminathan, Amber Meservey, Alice Parish, Cynthia L. Green, Kishan Parikh, Terry Fortin, Richard A. Krasuski, Jordan W. Whitson, Talal Dahhan, Yen-Rei Yu, Karla Kennedy, Susana Almeida-Peters, Sudarshan Rajagopal

**Affiliations:** a Department of Medicine, Duke University Medical Center, Durham, North Carolina; b Duke Clinical Research Institute, Durham, North Carolina; c Department of Medicine, University of Pennsylvania, Philadelphia, Pennsylvania; d Department of Biostatistics and Bioinformatics, Duke University Medical Center, Durham, North Carolina; e University of Colorado, Aurora, Colorado

**Keywords:** pulmonary arterial hypertension, prostacyclin, World Symposium on Pulmonary Hypertension, pulmonary vasodilator, treprostinil

## Abstract

**Background:**

Patients with pulmonary arterial hypertension (PAH) and additional cardiac or pulmonary comorbidities have a poor prognosis and are frequently excluded from clinical trials. The purpose of this study was to evaluate outcomes of patients with pulmonary hypertension (PH) secondary to a range of World Symposium on PH (WSPH) groups treated with inhaled treprostinil (iTRE) in a real-world setting.

**Methods:**

Patients with PH who were started on treatment with iTRE at Duke University were classified by WSPH Group and included patients with Groups 1, 2, 3, combined Groups 2 and 3 (PH in the setting of left heart failure and chronic lung disease), Group 4, and Group 5 PH. Time to disease worsening, a composite of death, lung transplantation, or transition to intravenous prostacyclin was compared by WSPH Group, and iTRE treatment status using a multivariable Cox proportional hazards model adjusted for age, sex, and Registry to Evaluate Early and Long-Term PAH Disease Management Lite 2 risk score. Treatment with iTRE was defined as a time-varying covariate.

**Results:**

The cohort included 270 patients with PH: 30.6% Group 1; 10% Group 2; 32.2% Group 3; 11.1% combined Groups 2 and 3; and 15.9% with either Group 4 or 5 PH. At 3 and 6 months of follow-up, 24.8% and 38.9% of patients, respectively, were no longer treated with iTRE. Patients who discontinued treatment with iTRE had a significantly higher risk of disease worsening (adjusted hazard ratio: 5.02, 95% confidence interval: 3.44-7.31). There was no significant difference in disease worsening among WSPH Groups.

**Conclusions:**

In a real-world setting, many patients with PH secondary to a range of WSPH Groups tolerated treatment with iTRE. Future studies should phenotype patients with PH based on both comorbidities and therapeutic responsiveness.

## Background

Pulmonary hypertension (PH) is increasingly identified in elderly patients with multiple comorbidities, raising the complexity of both diagnosis and management.^1^, ^2^ For example, older patients may be more likely to have left heart or chronic lung disease, leading to diagnostic uncertainty about the cause of PH, and in some cases, possible involvement of multiple World Symposium on Pulmonary Hypertension (WSPH) Groups.^3^, ^4^ Notably, there is a lack of evidence-based criteria for the expected degree of PH in left heart or lung disease,^5^, ^6^ and some patients appear to have a severe pulmonary vascular disease phenotype relative to the degree of underlying heart or lung disease. Prior studies suggest that patients with Group 1 PH with cardiac or pulmonary comorbidities are less likely to be treated with combination pulmonary vasodilator therapies^7^ and have a worse prognosis.^2^, ^8^, ^9^, ^10^ WSPH Group 1 patients with comorbidities are also frequently excluded from clinical trials and data on the effectiveness of pulmonary vasodilator therapy in this subset of patients are limited.

Treprostinil is a prostacyclin analog that inhibits platelet aggregation and promotes direct vasodilation of pulmonary and systemic arterial vascular beds.^11^ The inhaled formulation of treprostinil (inhaled treprostinil, [iTRE]) was initially approved for patients with Group 1 PH based on an improved 6-minute walk distance (6MWD) after 12 weeks.^12^ More recently, iTRE has been approved to treat PH in the context of interstitial lung disease (PH-ILD), making it the first pulmonary vasodilator to demonstrate benefit in a subset of WSPH Group 3 disease.^13^ However, even before the approval of iTRE for PH-ILD, many PH centers across the United States reported using pulmonary vasodilator therapy to treat WSPH Group 2 or 3 patients with a severe pulmonary vascular phenotype.^14^ Prior case series have described favorable short-term changes in such patients treated with iTRE.^15^, ^16^, ^17^, ^18^ However, little is known about real-world long-term drug tolerability or outcomes of patients with PH and concomitant heart or lung disease who are treated with iTRE.

The purpose of this study was to evaluate drug tolerability and outcomes of patients with PH secondary to a range of WSPH Groups treated with iTRE in a real-world setting. We further sought to characterize differences in disease progression among WSPH Groups and to determine if the association of iTRE treatment status and disease progression differed by WSPH Group.

## Methods

### Study design and population

This was a single-center retrospective cohort study that included all adults evaluated at Duke University Medical Center and started on treatment for PH with iTRE between August 26, 2009 and December 31, 2017. Titration protocols for iTRE at our center have been previously described.^16^ Briefly, patients were started on 3 breaths (18 µg) 4 times daily, followed by a trial of 6 breaths (36 µg) 4 times daily. Following this, patients were instructed to increase the dosage by 1 breath daily up to a maximum of 12 breaths (72 µg) 4 times daily as tolerated. This protocol was modified as clinically indicated for patients to ensure tolerability, either with a slower up-titration or to a lower target for maximum number of breaths. For patients who discontinued iTRE treatment, the reason for discontinuation was identified.

All patients had a mean pulmonary artery pressure (mPAP) ≥25 mm Hg and pulmonary vascular resistance (PVR) ≥3 Wood Units (WU) and were started on iTRE at the discretion of the treating physician. Patients were categorized according to the following PH cohorts: Groups 1, 2, 3, combined Groups 2 and 3, and Group 4 or 5. All patients with Group 2 PH also had a PVR ≥3 WU (combined postcapillary and precapillary PH) accompanied by features of left heart disease per the treating clinician, including a pulmonary capillary wedge pressure (PCWP) ≥15 mm Hg, valvular heart disease, heart failure with preserved ejection fraction, or heart failure with reduced ejection fraction. All patients with Group 3 PH had a PVR ≥3 WU, PCWP <15 mm Hg, and a forced vital capacity (FVC) ≤70% predicted along with evidence of parenchymal disease on high-resolution computed tomography imaging or a Forced Expiratory Volume in 1 second (FEV1)/FVC ratio <70% predicted. We did not differentiate between limited and severe chronic lung disease in the cohort of patients with Group 3 PH as it would require grading of the degree of parenchymal lung disease.^5^ Patients classified as combined Groups 2 and 3 PH met the criteria for Group 2 PH in the setting of chronic lung disease. All data were collected by retrospective chart abstraction from the electronic health record. This study was approved through the Duke University institutional review board (Pro00091555). The need for consent was waived by the ethics committee. The data were accessed for research purposes between January 1, 2019 and January 15, 2020.

### Study outcome and variable definitions

The primary outcome of this study was a composite measure of disease progression that included death, lung transplant, or transition to intravenous prostacyclin. Time to disease progression was calculated from the date of iTRE initiation to the earliest of the following events: disease progression event, last known date of clinical follow-up, or censor date of December 31, 2018. A sensitivity analysis was performed for the end-point of disease progression that included only death and lung transplant. Secondary outcome measures described were changes in 6MWD, FVC, diffusing capacity for carbon monoxide (DLCO), N-terminal pro–B-type natriuretic peptide (NT-proBNP), and right ventricular systolic pressure (RVSP) over the first year of follow-up after iTRE initiation.

The variables of interest were iTRE treatment status, PH cohort, and the interaction between PH cohort and iTRE treatment status. Patient’s iTRE treatment status was defined as a time-varying covariate, where a 7-day buffer between discontinuation of iTRE and an event was required for a subject to be considered as not taking iTRE at the time of an event. If an event occurred fewer than 7 days from discontinuation of iTRE, subjects were considered to be taking iTRE at the time of the event. All patients who transitioned to intravenous prostacyclin were considered to be taking iTRE at the time of the event. A 2-day buffer between discontinuation of iTRE and an event was also performed as a sensitivity analysis.

The covariates included age, sex, and the Registry to Evaluate Early and Long-Term Pulmonary Arterial Hypertension (PAH) Disease Management (REVEAL) Lite 2 risk score. The REVEAL Lite 2 is an abridged version of the REVEAL 2.0 risk calculator and includes the following variables: New York Heart Association or World Health Organization functional class (WHO-FC), systolic blood pressure, heart rate, 6MWD, NT-proBNP, and renal insufficiency.^19^ The REVEAL Lite 2 score was calculated at the time of iTRE initiation and missing variables were scored as zero, consistent with prior work.^19^ In patients who were hospitalized at the time of iTRE initiation, the 6MWD was considered to be 0 m.

### Statistical analysis

Descriptive statistics are presented as medians with 25th and 75th percentiles (Q1, Q3) for continuous variables and frequencies with percentages for categorical variables, and are used to summarize clinical characteristics at the time of iTRE initiation. We compared clinical characteristics for patients who remained on iTRE for at least 3 months to those who discontinued iTRE before 3 months using the Wilcoxon rank sum test or chi-square for continuous and categorical variables, respectively. The change in secondary outcomes 6MWD, NT-proBNP, RVSP, FVC % predicted, and DLCO % predicted from iTRE initiation to 3, 6, and 12 months is also presented descriptively.

To evaluate the association between iTRE treatment status and disease progression, we used a multivariable Cox proportional hazards regression model. Additionally, we assessed the Kaplan-Meier curve by iTRE discontinuation by 3 months (yes/no) groups using the log-rank test. To evaluate the association between PH cohorts and time to disease progression, we used a multivariable Cox proportional hazards regression model. Additionally, the Kaplan-Meier estimator was used to estimate the cumulative probability of disease progression as a function of time by WSPH Group. Lastly, we performed an interaction analysis to evaluate if the association between iTRE treatment status and disease progression differed by WSPH group. Sensitivity analyses were performed limiting the cohort to only subjects with complete information for the REVEAL Lite 2 risk calculator.

All analyses were performed using SAS Software version 9.4 (SAS Institute, Cary, NC). The proportional hazards assumption was checked using Schoenfeld residuals and was not found to be violated for any of the variables in the model.

## Results

### Patient characteristics

This study cohort included 270 patients with PH who were initiated on iTRE between 2009 and 2017. Patients were followed for a median (Q1, Q3) of 866 (365, 1,635) days after iTRE initiation. Patients were titrated up to a median iTRE dose of 12.0 breaths (9.0, 12.0) 4 times daily. The median age of the cohort was 64.0 years (53.0, 70.0) and the majority were female (68.9%) (Table 1). Patients were classified into different PH cohorts based on their WSPH group: 31.0% were group 1 (*n* = 83); 10.0% group 2 (*n* = 27); 32.0% group 3 (*n* = 87); 11.0% combined groups 2 and 3 (*n* = 30); and 16.0% were in either group 4 or 5 (*n* = 43) (Table 1). ILD was present in 93/270 (34.4%) patients and connective tissue disease was present in 78/270 (28.9%) patients. Most patients had severe PH at the time of iTRE initiation, evidenced by a median mPAP of 50.0 mm Hg (44.0, 57.0), median PVR of 9.1 WU (7.1, 11.6), and the majority (79.3%) being classified as WHO-FC III or IV (Table 1). At the time of iTRE initiation, 77/270 patients (28.5%) were being treated with an endothelin receptor antagonist (ERA), 141/270 (52.2%) were treated with a phosphodiesterase-5 inhibitor (PDE-5i), and 44/270 (16.3%) were on combination therapy with both an ERA and PDE-5i.

**Table 1:** Patient Characteristics Stratified by Time on Inhaled Treprostinil (iTRE)

Characteristics^a^	Overall cohort	Continued iTRE ≥3 months	Treated with iTRE <3 months^b^	*p*-value^c^
*N*	*270*	*203*	*67*	
*Demographics*				
Age (years)	64.0 (53.0, 70.0)	63.0 (53.0, 70.0)	64.0 (53.0, 73.0)	0.18
Female	186 (68.9%)	137 (67.5%)	49 (73.1%)	0.39
*PH classification and severity*				
WSPH Group				0.26
Group 1	83 (30.6%)	68 (33.5%)	15 (22.4%)	
Group 2	27 (10.0%)	19 (9.4%)	8 (11.9%)	
Group 3	87 (32.2%)	61 (30.0%)	26 (38.8%)	
Group 2 and 3^d^	30 (11.1%)	25 (12.3%)	5 (7.5%)	
Groups 4 or 5	43 (15.9%)	30 (14.8%)	13 (19.4%)	
WHO functional class				0.05
Group II	55 (20.4%)	49 (24.1%)	6 (9.0%)	
Group III	150 (55.6%)	108 (53.2%)	42 (62.7%)	
Group IV	64 (23.7%)	45 (22.2%)	19 (28.4%)	
6MWD (m)	240.5 (160, 348)^e^	246 (161, 351)	214 (155, 305)	0.27
NT-proBNP (pg/ml)	1,392 (528, 3336)^e^	1,431 (509, 3469)	1,300 (537, 3009)	0.65
mRAP (mm Hg)	11.0 (8.0, 15.0)	11.0 (8.0, 15.0)	10.0 (7.0, 14.5)	0.15
mPAP (mm Hg)	50.0 (44.0, 57.0)	51.0 (46.0, 58.0)	48.0 (42.0, 54.0)	0.05
PCWP (mm Hg)	12.5 (9.0, 17.0)	13.0 (9.0, 17.0)	12.0 (9.0, 15.0)	0.64
Cardiac Index (liter/min/m^2^)	2.1 (1.8, 2.5)	2.1 (1.8, 2.5)	2.2 (1.8, 2.6)	0.15
PVR (WU)	9.1 (7.1, 11.6)	9.3 (7.3, 11.8)	8.5 (6.3, 10.9)	0.09
REVEAL Lite 2^f^	3.0 (2.0, 4.0)	3.0 (2.0, 4.0)	3.0 (2.0, 4.0)	0.25
RVSP (mm Hg)	75.0 (62.0, 87.0)^e^	74.0 (62.0, 88.5)	79.0 (63.0, 86.0)	0.49
RV size^g^				0.30
Normal	21 (8.5%)	15 (8.1%)	6 (9.7%)	
Mildly enlarged	47 (19.0%)	32 (17.2%)	15 (24.2%)	
Moderately enlarged	109 (44.0%)	88 (47.3%)	21 (33.9%)	
Severely enlarged	71 (28.6%)	51 (27.4%)	20 (32.3%)	
LV hypertrophy^g^				0.24
Normal	90 (36.9%)	72 (39.8%)	18 (28.6%)	
Mild	107 (43.9%)	78 (43.1%)	29 (46.0%)	
Moderate	41 (16.8%)	28 (15.5%)	13 (20.6%)	
Severe	6 (2.5%)	3 (1.7%)	3 (4.8%)	
*Medical history*				
Connective tissue disease	78 (28.9%)	63 (31.0%)	15 (22.4%)	0.18
Obstructive airway disease	98 (36.3%)	72 (35.5%)	26 (38.8%)	0.62
Interstitial lung disease	93 (34.4%)	71 (35.0%)	22 (32.8%)	0.75
Sarcoidosis	28 (10.4%)	19 (9.4%)	9 (13.4%)	0.34
Obesity (BMI >30 kg/m^2^)	30 (11.1%)	22 (10.8%)	8 (11.1%)	0.80
Chronic kidney disease^h^	28 (10.4%)	25 (12.3%)	3 (4.5%)	0.07
Coronary artery disease	51 (18.9%)	39 (19.2%)	12 (17.9%)	0.81
Atrial fibrillation	30 (11.1%)	20 (9.9%)	10 (14.9%)	0.25
Obstructive sleep apnea	65 (24.1%)	52 (25.6%)	13 (19.4%)	0.30
Venous thromboembolism	30 (11.1%)	20 (9.9%)	10 (14.9%)	0.25
*Medications*				
ERA	77 (28.5%)	63 (31.0%)	14 (20.9%)	0.11
Ambrisentan	17 (6.3%)	14 (6.9%)	3 (4.5%)	
Bosentan	49 (18.1%)	40 (19.7%)	9 (13.4%)	
Macitentan	11 (4.1%)	9 (4.4%)	2 (3.0%)	
PDE-5i	141 (52.2%)	104 (51.2%)	27 (55.2%)	0.57
Sildenafil	65 (24.1%)	49 (24.1%)	16 (23.9%)	
Tadalafil	76 (28.1%)	55 (27.1%)	21 (31.3%)	
Riociguat	2 (0.7%)	1 (0.5%)	1 (0.5%)	0.41
Combination of ERA and PDE-5i	44 (16.3%)	35 (17.2%)	9 (13.4%)	0.46
*Other characteristics*				
FVC (% predicted)	65.0 (49.0, 81.0) N = 199	64.5 (49.7, 85.0) N = 146	65.0 (47.0, 74.0) N = 53	0.31
DLCO (% predicted)	32.0 (24.0, 50.0) N = 171	34.0 (25.0, 52.0) N = 127	30.0 (23.5, 44.0) N = 44	0.20
Maximum iTRE dose (number of breaths)	12.0 (9.0, 12.0)	12.0 (12.0, 12.0)	8.0 (6.0, 11.0)	<0.001
Time treated with iTRE (months)	12.0 (3.0, 29.0)	18.0 (9.0, 36.0)	1.0 (0.5, 2.0)	<0.001

Abbreviations: 6MWD, 6-minute walk distance; BMI, body mass index; DLCO, diffusing capacity of carbon monoxide; ERA, endothelin receptor antagonist; FVC, forced vital capacity; HR, hazard ratio; iTRE, inhaled treprostinil; LV, left ventricular; mPAP, mean pulmonary artery pressure; mRAP, mean right atrial pressure; NT-proBNP, N-terminal pro–B-type natriuretic peptide; PCWP, pulmonary capillary wedge pressure; PDE-5i, phosphodiesterase-5 inhibitor; PH, pulmonary hypertension; PVR, pulmonary vascular resistance; REVEAL, Registry to Evaluate Early and Long-Term Pulmonary Arterial Hypertension Disease Management; RV, right ventricular; RVSP, right ventricular systolic pressure; WHO, World Health Organization; WSPH, World Symposium on Pulmonary Hypertension; WU, Wood Units.

Data are median (25th percentile [Q1]-75th percentile [Q3]) or N (%).

a Assessed before iTRE start.

b Reasons for not remaining on iTRE for at least 3 months include death, loss of follow-up, and treatment discontinuation.

c Wilcoxon rank sum test used for continuous variables, and chi-square test used for categorical variables.

d PH in the setting of left heart failure and chronic lung disease.

e Data were missing for 10% to 20% of patients.

f REVEAL Lite includes the following variables: renal insufficiency, WHO functional class, vital signs (systolic blood pressure, HR), 6MWD, NT-proBNP.

g Right ventricular size and left ventricular hypertrophy as measured on echocardiography.

h Defined as glomerular filtration rate of <60 ml/min/1.73 m^2^ for ≥3 months.

Clinical and hemodynamic characteristics differed significantly by PH cohort (Supplementary Table 1). Patients with Group 1 had the largest proportion of patients classified as WHO-FC II (30.1%) and the largest 6MWD (309.6 vs 230 m in Group 3). Group 2 PH and combined Groups 2 and 3 PH patients had the highest median PCWP (19.0 [17.0, 23.0] and 19.5 [17.0, 21.0], respectively). Most patients with Group 3 PH (60/87, 69.0%) had ILD and 49/87 (56.3%) had airway obstruction.

At 3-month follow-up, 67 patients (24.8%) were no longer taking iTRE, due to either death (15/67, 22.4%), loss of follow-up (9/67, 13.4%), or treatment discontinuation (44/67, 65.7%, Table 2). The most frequent reasons for treatment discontinuation included dyspnea or hypoxemia, lack of clinical improvement, or other side effects (Table 2). At 6-month follow-up, 105 patients (38.9%) were no longer taking iTRE (Table 2). Clinical characteristics were similar among patients who continued iTRE for at least 3 months vs those who did not (Table 1). Patients who discontinued iTRE before 3 months had a slightly lower median mPAP compared to patients who remained on iTRE ≥3 months (48.0 [42.0, 54.0] vs 51.0 [46.0, 58.0], *p* = 0.047) though were numerically more likely to be classified as WHO-FC III or IV (91.9% vs 75.4%, *p* = 0.054). A higher proportion of patients with groups 2, 3, and 4 or 5 PH were no longer taking iTRE at 3 months (29%, 30%, and 30%, respectively) compared with group 1 and combined groups 2 and 3 PH (18% and 17%, respectively), though this difference was not statistically significant (*p* = 0.26).

**Table 2:** iTRE Treatment Status and Reasons for Discontinuation at 3 and 6 Months

Status at time point	3 months	6 months^a^
Continued on iTRE	203 (75.2%)	165 (61.1%)
Death	15 (5.6%)	30 (11.1%)
Lost to follow-up	8 (3.0%)	17 (6.3%)
Discontinued	44 (16.3%)	58 (21.5%)
Reason for discontinuation^b^		
Dyspnea or hypoxemia	19 (43.2%)	19 (32.8%)
No improvement	8 (18.2%)	10 (17.2%)
Headache	7 (15.9%)	10 (17.2%)
Nausea/vomiting or abdominal pain	5 (11.4%)	7 (12.1%)
Cough	3 (6.8%)	4 (6.9%)
Chest pain	2 (4.5%)	2 (3.4%)
Cost	1 (2.3%)	2 (3.4%)
Transition to IV prostacyclin	0 (0%)	2 (3.4%)
Other	1 (2.3%)	8 (13.8%)

Abbreviation: iTRE, inhaled treprostinil.

a Six months are cumulative of 3 and 6 months.

b Percentages are reported among those who discontinued.

### Association of iTRE treatment status with disease progression

During the study period, a total of 164/270 patients (60.7%) experienced disease progression, as defined by either death (*n* = 146/270, 54.3%), lung transplant (*n* = 2/270, 0.2%), or transition from iTRE to an intravenous prostacyclin (*n* = 16/270, 5.6%). Patients who discontinued iTRE before 3 months had a significantly shorter time to disease progression (log-rank *p* = 0.009, Supplemental Figure 1). In a multivariable analysis adjusted for age, sex, and REVEAL Lite 2 score, patients who discontinued treatment with iTRE more than 7 days before an event had a significantly higher risk of disease progression (adjusted hazard ratio [HR] = 5.02; 95% confidence interval [CI]: [3.44, 7.31]; *p* < 0.001; Table 3) compared to patients who either remained on iTRE or had an event within 7 days of iTRE discontinuation. Results were similar in a sensitivity analysis that included only the 194 patients who had complete information for the REVEAL Lite 2 score (adjusted HR = 5.97; 95% CI: [3.72, 9.57]; *p* < 0.001). An additional sensitivity analysis that compared the risk of disease worsening in patients who discontinued iTRE at least 2 days before an event with patients who either remained on iTRE or had an event within 2 days of stopping iTRE was also similar (HR = 9.16; 95% CI: [6.01, 13.98]; *p* < 0.001).

**Table 3:** Hazard Ratio Estimates for the Time to Composite End-point of Death, Lung Transplant, or Transition to Intravenous Prostacyclin, Assessing iTRE Treatment Status

Characteristics	Disease progression^a^^,^^b^	
	HR (95% CI)	*p*-value
Age	1.00 (0.98, 1.01)	0.96
Female	0.55 (0.40, 0.76)	<0.001
REVEAL Lite 2 score	1.31 (1.19, 1.44)	<0.001
Discontinuation of iTRE^c^	5.02 (3.44, 7.31)	<0.001

Abbreviations: CI, confidence interval; HR, hazard ratio; iTRE, inhaled treprostinil; REVEAL, Registry to Evaluate Early and Long-Term Pulmonary Arterial Hypertension Disease Management.

a Disease progression is defined as a composite of death, lung transplant, or transition to intravenous prostacyclin.

b Evaluated using multivariable Cox proportional hazards regression model.

c Defined as discontinuing iTRE treatment at least 7 days before a disease progression event. Patients who transitioned to IV prostacyclin were considered as taking iTRE at the time of event.

Of the 16 patients who were transitioned to parenteral prostacyclin therapy, 11 subsequently died and 1 patient underwent lung transplantation. A sensitivity analysis that did not include transition to parenteral prostacyclin therapy in the disease progression end-point demonstrated similar results (Supplementary Table 2).

### Association of WSPH Groups with disease progression

**Figure 1:**
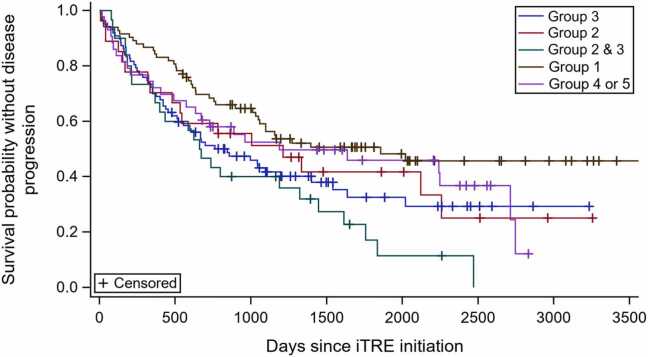
Time to a composite of death, lung transplant, or transition to IV prostacyclin, stratified by WSPH group. iTRE, inhaled treprostinil; WSPH, World Symposium on Pulmonary Hypertension.

During the study period, 42/83 (50.6%) patients with group 1 PH experienced a disease progression event compared with 17/27 (63.0%) patients with group 2 PH, 54/87 (62.1%) patients with group 3 PH, 25/30 (83.3%) patients with combined groups 2 and 3 PH, and 46/43 (60.5%) with group 4 or 5 PH (log-rank *p* = 0.020, Figure 1). In a multivariable analysis adjusted for age, sex, and REVEAL Lite 2 score, there was no significant association between PH cohort and disease progression (*p* = 0.18, Table 4). While not statistically significant, patients with combined groups 2 and 3 PH had a numerically increased risk of disease progression compared to patients classified as group 1 in multivariable analysis (HR = 1.82, 95% CI: [1.09, 3.04], Table 4). Results were consistent in sensitivity analyses that (1) included only patients with complete data to calculate REVEAL Lite 2 scores (data not shown) and (2) excluded transition to parenteral prostacyclin therapy from the disease progression end-point (Supplementary Table 3).

**Table 4:** Hazard Ratio Estimates for the Time to Composite End-point of Death, Lung Transplant, or Transition to Intravenous Prostacyclin Assessing the WSPH Groups

Characteristics	Disease progression^a^,^b^	
	HR (95% CI)	*p*-value
Age	1.00 (0.98, 1.01)	0.62
Female	0.64 (0.46, 0.89)	0.01
REVEAL Lite 2 score	1.27 (1.15, 1.40)	<0.001
WSPH group		0.18
Group 2 vs group 1	1.51 (0.85-2.68)	
Group 3 vs group 1	1.40 (0.92-1.12)	
Groups 2 and 3 vs group 1	1.82 (1.09-3.04)	
Group 4 or 5 vs group 1	1.16 (0.70-1.92)	

Abbreviations: CI, confidence interval; HR, hazard ratio; REVEAL, Registry to Evaluate Early and Long-Term Pulmonary Arterial Hypertension Disease Management; WSPH, World Symposium on Pulmonary Hypertension

a Disease progression is defined as a composite of death, lung transplant, or transition to intravenous prostacyclin.

b Evaluated using multivariable Cox proportional hazards regression model.

### Interaction between iTRE discontinuation and WSPH Groups/comorbidities on disease progression

In a multivariable Cox proportional hazards model, after adjusting for age, sex, and REVEAL Lite 2 score, there was no significant interaction between iTRE treatment status and PH cohort on disease worsening (interaction *p*-value = 0.11) (Supplementary Table 4).

### Change in secondary clinical outcomes from baseline

**Figure 2:**
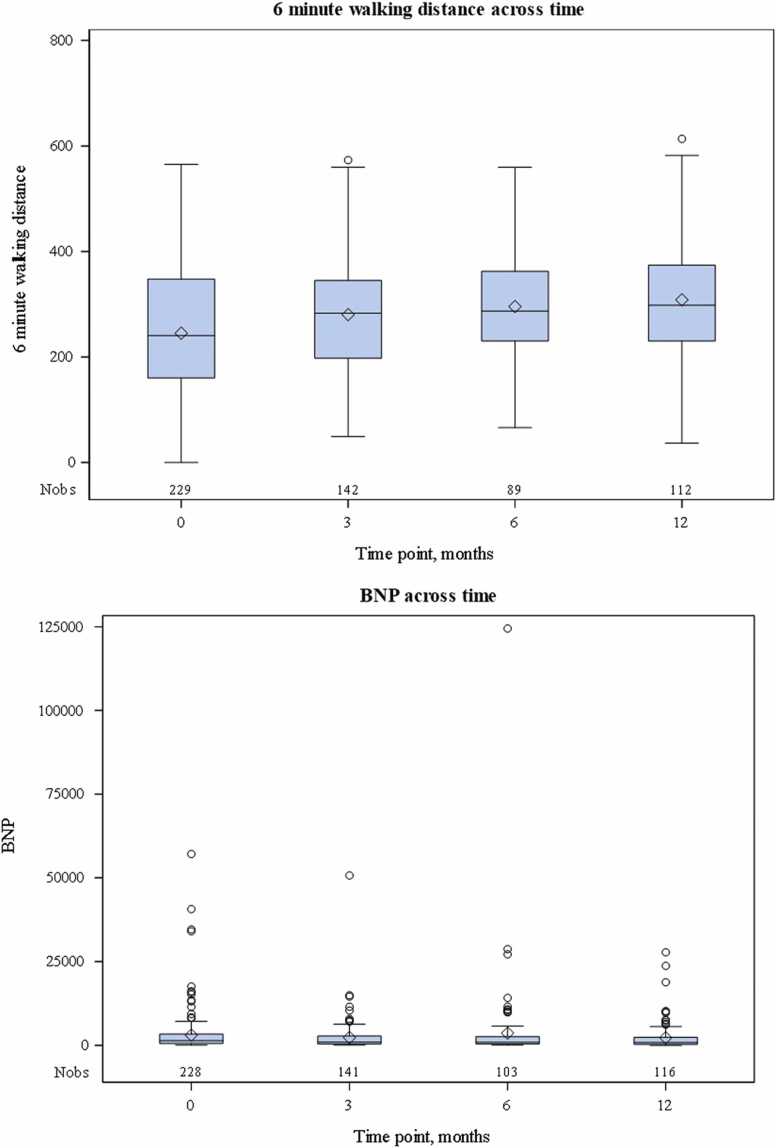
Changes in 6-minute walking distance (top) and NT-proBNP (bottom) following iTRE initiation. BNP, B-type natriuretic peptide; iTRE, inhaled treprostinil; NT-proBNP, N-terminal pro–B-type natriuretic peptide.

The median (Q1, Q3) improvement in 6MWD in the overall cohort after iTRE initiation was 14.7 m (−29.5, 79.6) at 3 months, 26.2 m (−13.3, 95.6) at 6 months, and 33.3 m (−19.0, 93.4) at 12 months (Table 5, Figure 2: changes in 6MWD (top) and NT-proBNP (bottom) following iTRE initiation). The median (Q1, Q3) change in NT-proBNP in the overall cohort after iTRE initiation was −207 (−1134, 158) at 3 months, −208 (−1079.5, 300) at 6 months, and −164 (−961, 351) at 12 months (Table 5, Figure 2). Changes in 6MWD and NT-proBNP were comparable when stratified by WSPH group (Supplementary Table 5). Evaluation of other clinical measures, including FVC, DLCO, and RVSP, was limited by missing data.

**Table 5:** Change in Clinical Characteristics After iTRE Initiation

Characteristics	Time after iTRE initiation		
	3 months	6 months	12 months
6MWD (m)	14.7 (−29.5, 79.6) N = 123^a^	26.2 (−13.3, 95.6) N = 80^b^	33.3 (−19.0, 93.4) N = 98^b^
NT-proBNP (pg/ml)	−207 (−1134, 158) N = 122^a^	−208 (−1079.5, 300) N = 92^b^	−164 (−961, 351) N = 95^b^

Abbreviations: 6MWD, 6-minute walk distance; iTRE, inhaled treprostinil; NT-proBNP, N-terminal pro–B-type natriuretic peptide.

Data are median (25th percentile [Q1]-75th percentile [Q3]).

a About 95% of patients were persisting with iTRE treatment.

b About 99% of patients were persisting with iTRE treatment.

## Discussion

Our study demonstrates that in a real-world cohort of patients with PH secondary to a range of WSPH Groups, treatment with iTRE was tolerated by the majority of patients. Clinical characteristics among patients who continued iTRE for at least 3 months vs those who did not continue the medication were similar. While unadjusted analysis demonstrated that the risk of disease progression was highest among patients with combined Groups 2 and 3 PH, there was no significant difference in disease progression among WSPH Groups after adjustment for PH disease severity measures.

PH patients increasingly have comorbidities that make their placement in the current WSPH clinical classification system difficult. For example, in the PVDOMICS study, 38.9% of patients with PH had “mixed PH” that could be described by primary and secondary WSPH groups.^20^ This reflects clinical practice, where patients frequently fall into multiple WSPH groups, with PH patients having significant comorbidities including heart and lung disease.^21^ During the time when these patients were being treated (2009-2017), iTRE was only approved for the treatment of Group 1 PH,^12^ as its efficacy in Group 3 PH related to ILD was only later demonstrated in the INCREASE trial.^13^ It is likely that nearly all of the patients in this study were felt to have a significant precapillary, that is, Group 1, component to their disease, as physicians had decided on treatment with iTRE. This is also consistent with the hemodynamics of the overall cohort, with a mPAP of 50 and a mean PVR of 9.1 (Table 1).

While most patients in this study had severe precapillary PH, relatively few patients were treated with a combination ERA and PDE-5i vasodilator therapy at the time of iTRE initiation. This is in part related to the limited evidence for pulmonary vasodilator therapy, and particularly for combination therapy, in patients who meet the criteria for Group 2 or 3 PH. Patients in the current study were likely initiated on treatment with iTRE given the severity of precapillary PH and suspicion that symptoms were driven by right heart failure rather than other comorbidities. Importantly, at 3 months after iTRE medication initiation, approximately 75% of patients persisted with iTRE treatment by 3 months and 61% persisted with iTRE by 6 months after initiation. This is similar to a recent claims data analysis of patients with PAH, which noted the persistence of iTRE treatment at 6 months to be 65%,^22^ suggesting there may not be a large difference in tolerability based on cardiac or pulmonary comorbidities.

Prior studies have demonstrated better survival in patients with Group 1 PH compared with other WSPH Groups, particularly patients with Group 2 or 3 PH,^23^, ^24^ as well as better survival in patients with PAH who do not have additional cardiac or pulmonary comorbidities.^2^, ^8^ Our unadjusted analysis is also consistent with this prior work, as the highest risk of disease progression was identified in patients with combined Groups 2 and 3 PH and the lowest risk in patients with isolated Group 1 PH. However, this difference was attenuated and no longer statistically significant after adjustment for demographics and PH severity measures. Moreover, the association of iTRE treatment status and disease progression did not differ by WSPH Group. Our results support the concept that the complexity of PH is not always captured through the current clinical classification system. Regardless of comorbidities, there may be a subset of patients who are responsive to pulmonary vasodilators and benefit from iTRE treatment. Ongoing efforts through studies, such as PVDOMICS, may better phenotype patients with PH based on pathophysiology and allow for more insights into therapy selection.

This study has several limitations. First, all patients were started on iTRE at a tertiary care PH care center after a thorough evaluation, and this approach should not be generalized without additional research. Current guidelines do not support routine use of pulmonary vasodilator therapy in patients with Group 2 PH or Group 3 PH related to chronic obstructive pulmonary disease. Second, this retrospective cohort study cannot evaluate a causal relationship between iTRE discontinuation and PH disease progression; more likely this subgroup experienced disease worsening despite vasodilator trials. To minimize bias, a conservative 7-day buffer between iTRE discontinuation and clinical event was applied, though the ideal comparator arm would be patients with severe precapillary PH and significant comorbidities who were not initiated on iTRE. Third, while this is one of the largest retrospective studies of patients with PH treated with iTRE, our power to detect differences in the association of iTRE and disease progression in separate WSPH groups/comorbidities is still limited. Finally, follow-up data were collected based on routine clinical care, and systematic echocardiographic, pulmonary function testing, and hemodynamic follow-up data were lacking.

In conclusion, this study supports the long-term tolerability of iTRE in patients with a broad range of WSPH groups, including Groups 2 and 3 PH. Additional prospective studies are needed to better phenotype patients with PH based on both comorbidities and therapeutic responsiveness.

## Ethical approval

This study was approved through the Duke University institutional review board (Pro00091555).

## Disclosure statement

Sudar Rajagopal reports financial support was provided by United Therapeutics Corporation Research and Development. Aparna Swaminathan reports a relationship with United Therapeutics Corporation Research and Development that includes consulting or advisory. Sudar Rajagopal reports a relationship with United Therapeutics Corporation Research and Development that includes consulting or advisory and funding grants, Apie Therapeutics that includes consulting or advisory, Altavant Sciences, Inc. that includes consulting or advisory, Gossamer Bio that includes consulting or advisory, Insmed Inc. that includes consulting or advisory, Janssen Pharmaceuticals Inc. that includes consulting or advisory and funding grants, Liquidia Corporation Inc. that includes consulting or advisory, and Polarean that includes consulting or advisory. Dr Rajagopal is also a patent-holder on US Patent 62/673,175 entitled “Dynamic 129Xe Gas Exchange Spectroscopy” that is licensed to Polarean and unrelated to the current work. The other authors declare that they have no known competing financial interests or personal relationships that could have appeared to influence the work reported in this paper.

This was presented as an oral presentation at Chest 2021.
