# Supplementary material for: Evaluation of patients with severe pulmonary hypertension and a range of comorbidities prescribed inhaled treprostinil
Source: JHLT Open. 2024 Jul 23;6:100131. doi: 10.1016/j.jhlto.2024.100131 (PMC11935515; doi:10.1016/j.jhlto.2024.100131)

**Supplemental Tables and Figures**

**Supplemental Table 1.** Patient characteristics stratified by WSPH Group

| **Characteristic** | **WSPH Group** | | | | | | |
| --- | --- | --- | --- | --- | --- | --- | --- |
|  | **Group 1**  N=83 | **Group 2**  N=27 | **Group 2 & 3**  N=30 | **Group 3**  N=87 | **Groups 4 or 5**  N=43 | **Total**  N=270 | **p-value** |
| Age (years) | 60.0 (47.0, 69.0) | 64.0 (55.0, 72.0) | 64.5 (55.0, 70.0) | 66.0 (55.0, 71.0) | 61.0 (54.0, 69.0) | 64.0 (53.0, 70.0) | 0.05^1^ |
| Female | 56 (67.5%) | 22 (81.5%) | 20 (66.7%) | 54 (62.1%) | 34 (79.1%) | 186 (68.9%) | 0.19^2^ |
| WHO Functional Class |  |  |  |  |  |  | 0.001^2^ |
| Group II | 25 (30.1%) | 6 (22.2%) | 7 (23.3%) | 10 (11.5%) | 7 (16.3%) | 55 (20.4%) |  |
| Group III | 50 (60.2%) | 18 (66.7%) | 12 (40.0%) | 46 (52.9%) | 24 (55.8%) | 150 (55.6%) |  |
| Group IV | 8 (9.6%) | 3 (11.1%) | 10 (33.3%) | 31 (35.6%) | 12 (27.9%) | 64 (23.7%) |  |
| 6MWD (meters) | 309.6 (184.8, 409.0) | 245.0 (139.9, 296.3) | 216.7 (124.4, 329.2) | 230.4 (164.6, 296.3) | 204.4 (131.9, 313.7) | 240.5 (160.0, 347.7) | 0.003^1^ |
| NT-proBNP (pg/mL) | 991.0 (359.0, 2625.0) | 1863.0 (971.0, 3630.0) | 2071.0 (1018.5, 4636.0) | 1363.0 (561.0, 3430.0) | 1568.0 (718.0, 3747.0) | 1392.0 (528.0, 3336.5) | 0.070^1^ |
| mRAP (mmHg) | 10.0 (6.0, 13.0) | 15.0 (12.0, 17.0) | 16.0 (12.0, 20.0) | 9.0 (7.0, 13.0) | 13.0 (9.0, 18.0) | 11.0 (8.0, 15.0) | <0.001^1^ |
| mPAP (mmHg) | 51.0 (46.0, 56.0) | 53.0 (50.0, 62.0) | 53.5 (50.0, 62.0) | 47.0 (40.0, 52.0) | 49.5 (44.0, 60.0) | 50.0 (44.0, 57.0) | <0.001^1^ |
| PCWP (mmHg) | 10.0 (8.0, 13.0) | 19.0 (17.0, 23.0) | 19.5 (17.0, 21.0) | 10.0 (8.0, 13.0) | 15.0 (10.0, 18.0) | 12.5 (9.0, 17.0) | <0.001^1^ |
| CI (L/min/m^2^) | 2.1 (1.8, 2.5) | 2.1 (1.7, 2.5) | 2.0 (1.8, 2.4) | 2.1 (1.8, 2.5) | 2.2 (1.9, 2.6) | 2.1 (1.8, 2.5) | 0.572^1^ |
| PVR (WU) | 10.1 (7.5, 13.3) | 8.7 (6.9, 11.5) | 8.9 (7.7, 11.4) | 8.5 (7.0, 10.9) | 8.3 (6.5, 12.7) | 9.1 (7.1, 11.6) | 0.130^1^ |
| REVEAL Lite 2^3^ | 2.0 (1.0, 3.0) | 3.0 (2.0, 4.0) | 3.5 (2.0, 4.0) | 3.0 (2.0, 4.0) | 4.0 (3.0, 5.0) | 3.0 (2.0, 4.0) | <0.001^1^ |
| **Medical History** |  |  |  |  |  |  |  |
| Connective Tissue Disease | 20 (24.1%) | 8 (29.6%) | 14 (46.7%) | 34 (39.1%) | 2 (4.7%) | 78 (28.9%) | <0.001^2^ |
| Obstructive Airway Disease | 18 (21.7%) | 3 (11.1%) | 16 (53.3%) | 49 (56.3%) | 12 (27.9%) | 98 (36.3%) | <0.001^2^ |
| Interstitial Lung Disease | 13 (15.7%) | 2 (7.4%) | 18 (60.0%) | 60 (69.0%) | 0 (0.0%) | 93 (34.4%) | <0.001^2^ |
| Obesity | 10 (12.0%) | 4 (14.8%) | 3 (10.0%) | 8 (9.2%) | 5 (11.6%) | 30 (11.1%) | 0.936^2^ |
| Chronic Kidney Disease^4^ | 7 (8.4%) | 2 (7.4%) | 3 (10.0%) | 11 (12.6%) | 5 (11.6%) | 28 (10.4%) | 0.886^2^ |
| Coronary Artery Disease | 16 (19.3%) | 4 (14.8%) | 7 (23.3%) | 23 (26.4%) | 1 (2.3%) | 51 (18.9%) | 0.020^2^ |
| Atrial Fibrillation | 5 (6.0%) | 4 (14.8%) | 4 (13.3%) | 11 (12.6%) | 6 (14.0%) | 30 (11.1%) | 0.516^2^ |
| Obstructive Sleep Apnea | 17 (20.5%) | 4 (14.8%) | 12 (40.0%) | 19 (21.8%) | 13 (30.2%) | 65 (24.1%) | 0.128^2^ |
| **Medications** |  |  |  |  |  |  |  |
| ERA | 32 (38.6%) | 5 (18.5%) | 10 (33.3%) | 12 (13.8%) | 18 (41.9%) | 77 (28.5%) | 0.001^2^ |
| PDE-5i | 52 (62.7%) | 16 (59.3%) | 17 (56.7%) | 34 (39.1%) | 22 (51.2%) | 141 (52.2%) | 0.034^2^ |
| Combination of ERA & PDE-5i | 17 (20.5%) | 3 (11.1%) | 6 (20.0%) | 6 (6.9%) | 12 (27.9%) | 44 (16.3%) | 0.019^2^ |
| **Other Characteristics** |  |  |  |  |  |  | <0.001^1^ |
| FVC (% predicted) | 78.0 (68.0, 93.0) | 72.0 (61.0, 79.0) | 52.0 (44.0, 63.0) | 62.0 (45.0, 78.0) | 54.5 (44.0, 67.5) | 65.0 (49.0, 81.0) | <0.001^1^ |
| DLCO (% predicted) | 37.5 (25.0, 58.0) | 57.0 (46.0, 69.0) | 32.0 (29.0, 38.0) | 27.5 (22.0, 34.0) | 35.0 (26.0, 53.5) | 32.0 (24.0, 50.0) | <0.001^1^ |
| Data are median (25th percentile [Q1] - 75th percentile [Q3]) or N (%).  6MWD = six minute walk distance; DLCO = diffusing capacity of carbon monoxide; ERA = endothelin receptor antagonist; FVC = forced vital capacity; iTRE = inhaled treprostinil; mRAP = mean right atrial pressure; RVSP = right ventricular systolic pressure; WSPH = World Symposium on Pulmonary Hypertension  ^1^Evaluated using Kruskal Wallis test  ^2^Evaluated using Chi-Square test  ^3^REVEAL Lite includes the following variables: renal insufficiency, WHO functional class, vital signs (SBP, HR), 6MWD, NT-proBNP  ^4^Defined as glomerular filtration rate of <60ml/min/1.73m^2^ for ≥ 3 months | | | | | | | |

|  | **Disease Progression** ^a,b^ | |
| --- | --- | --- |
|  | **HR (95% CI)** | **p-value** |
| Age | 1.00 (0.99, 1.01) | 0.95 |
| Female | 0.51 (0.37, 0.71) | <0.001 |
| REVEAL Lite 2 score | 1.34 (1.22, 1.48) | <0.001 |
| Discontinuation of iTRE **^c^** | 5.67 (3.81, 8.43) | <0.001 |
| CI = confidence interval; HR = hazard ratio; iTRE = inhaled treprostinil; WSPH = World Symposium on Pulmonary Hypertension  **^a^** Disease progression is defined as a composite of death or lung transplant  **^b^** Evaluated using multivariable Cox proportional hazards regression model  ^c^ Defined as discontinuing iTRE treatment at least 7 days prior to a disease progression event. Patients who transitioned to IV prostacyclin were considered as taking iTRE at time of event. | | |

**Supplemental Table 2:** Hazard ratio estimates for the time to composite endpoint of death, or lung transplant, assessing iTRE treatment status

**Supplemental Table 3:** Hazard ratio estimates for the time to composite endpoint of death, or lung transplant, assessing WSPH groups

|  | **Disease Progression ^a^**^,b^ | |
| --- | --- | --- |
|  | **HR (95% CI)** | **P-Value** |
| Age | 1.00 (0.98, 1.01) | 0.62 |
| Female | 0.64 (0.46, 0.89) | 0.01 |
| REVEAL Lite 2 score | 1.27 (1.15, 1.40) | <0.001 |
| WSPH Group |  | 0.18 |
| Group 2 vs. Group 1 | 1.51 (0.85 – 2.68) |  |
| Group 3 vs. Group 1 | 1.82 (1.01 – 3.04) |  |
| Group 2 & 3 vs. Group 1 | 1.40 (0.92 – 2.12) |  |
| Groups 4 or 5 vs. Group 1 | 1.15 (0.70 – 1.92) |  |
| CI = confidence interval; HR = hazard ratio; iTRE = inhaled treprostinil; WSPH = World Symposium on Pulmonary Hypertension  **^a^** Disease progression is defined as a composite of death or lung transplant  **^b^** Evaluated using multivariable Cox proportional hazards regression model | | |

**Supplemental Table 4.** Hazard ratios of disease progression from multivariable Cox PH regression model, assessing the interaction between WSPH group and discontinuation of iTRE.

|  | **Disease Progression ^a^** | |
| --- | --- | --- |
|  | **HR (95% CI)** | **p-value** |
| Age | 1.00 (0.98, 1.01) | 0.665 |
| Female | 0.52 (0.37, 0.72) | <0.001 |
| REVEAL Lite 2 score | 1.30 (1.18, 1.43) | <0.001 |
| Discontinuation of iTRE **^b^** (main effect) | -- | <0.001 |
| WSPH group (main effect) | -- | 0.228 |
| WSPH group * Discontinuation of iTRE **^c^** (interaction) | -- | 0.110 |
| CI = confidence interval; HR = hazard ratio; iTRE = inhaled treprostinil; WSPH = World Symposium on Pulmonary Hypertension  **^a^** Disease progression is defined as a composite of death, lung transplant or transition to intravenous prostacyclin  **^b^** Defined as discontinuing iTRE treatment at least 7 days prior to a disease progression event  **^c^** Interaction analysis did not demonstrate a significant interaction between iTRE use and WSPH group, thus HRs WSPH group*discontinuation of iTRE are not reported. | | |

**Supplemental Table 5.** Change in clinical characteristics after iTRE initiation, stratified by WSPH Group

|  | **Group 1**  (N=83) | **Group 2**  (N=27) | **Group 3**  (N=87) | **Group 2 & 3**  (N=30) | **Groups 4 or 5**  (N=43) |
| --- | --- | --- | --- | --- | --- |
| **6MWD (m)** |  | | | | |
| **3 months** | 9.8 (-27.9, 87.3) N= 44 | 66.9 (16.5, 96.1)  N = 10 | -4.6 (-32.9, 34.4)  N = 33 | 16.9 (-30.1, 78.3)  N = 18 | 19.2 (-36.5, 79.5)  N =18 |
| **6 months** | 21.2 (-15.6, 97.9)  N=30 | 56.1 (39.3, 77.8)  N=5 | 16.5 (-20.1, 75.8)  N=25 | 10.5 (-3.8, 55.4)  N=10 | 47.9 (-16.4, 121.7)  N=10 |
| **12 months** | 40.2 (-10.0, 92.4)  N=37 | 60.9 (-0.4, 99.2)  N=8 | 1. (-61.2, 120.7)   N= 29 | 28.7 (12.4, 66.7)  N =10 | 44.8 (-6.4, 143.5)  N= 14 |
| **NT-proBNP (pg/mL)** |  | | | | |
| **3 months** | -95 (-459, 175), N=43 | -43 (-750, 110)  N= 11 | -377 (-1386, 171)  N=33 | -995 (-2014, 20)  N =16 | -337 (-1073, 1195) N=19 |
| **6 months** | -206 (-727, 429)  N=36 | -57 (-507, 977)  N=8 | -574 (-1606, 4)  N=23 | -733 (-1970, 109)  N=12 | -80 (-847, 505)  N= 13 |
| **12 months** | -312 (-815, 659)  N=34 | -164 (-742, 109)  N-7 | -133 (-1076, 248)  N=28 | -534 (-1351, 737)  N=12 | -383 (-1052, 408)  N=14 |

Data are median (25th percentile [Q1] - 75th percentile [Q3])

**Supplemental Figure 1:** Time to a composite of death, lung transplant, or transition to IV prostacyclin, stratified by duration of inhaled treprostinil use.


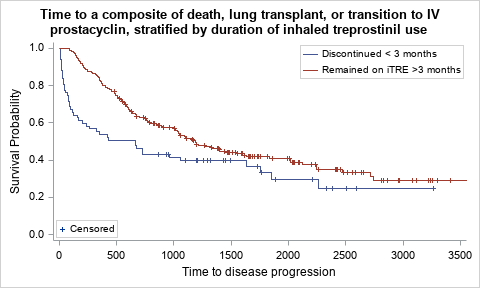

Supplement: Supplementary file 1 — Supplementary material [file mmc1.docx]
